# Dietary intake of methylmercury by 0–5 years children using the duplicate diet method in Japan

**DOI:** 10.1265/ehpm.24-00048

**Published:** 2024-05-10

**Authors:** Nozomi Tatsuta, Kaname Asato, Miyuki Iwai-Shimada, Kenta Iwai, Shoji F. Nakayama, Shin Yamazaki, Kunihiko Nakai

**Affiliations:** 1 Health and Environmental Risk Division, National Institute for Environmental Studies, Tsukuba 305-8506, Japan; 2 Development and Environmental Medicine, Tohoku University Graduate School of Medicine, Sendai 980-8575, Japan; 3 School of Sport and Health Science, Tokai Gakuen University, Miyoshi 470-0207, Japan

**Keywords:** Methylmercury, Dietary intake, 24-hour duplicate diet method, Children

## Abstract

**Background:**

The developing brains are sensitive to methylmercury (MeHg). However, the exposure to MeHg in baby foods and toddler meals remains unknown. This study aimed to determine MeHg intake from baby food or toddler meals, and to investigate the relationship with child hair total mercury (THg).

**Methods:**

A total of 3 days of 24-hour dietary diet and hair samples were collected from 260 consenting children aged 0–5 years. We measured the concentrations of THg and MeHg in the diet and THg in the hair.

**Results:**

The results of measuring THg were below both the method detection and method quantification limits or either of both in powdered milk (93.8%), 5–6 months (53.3%), and 7–8 months (39.5%). The median daily THg intake was 20.3 (95% confidence interval 0.72–232.5) ng/kgbw. MeHg was not detected in 213 samples with dietary THg concentrations below 1 ng/g. The MeHg concentration with THg concentrations of 1 ng/g or higher was 1.70 (0.87–6.21) ng/g, and MeHg percentage in THg was 90.0%. To estimate MeHg intake, we multiplied the THg concentration by 90.0%, resulting in an estimated MeHg intake of 18.3 (0.65–209.2) ng/kgbw/day. The THg in children’s hair was 1.05 (0.31–3.96) ppm, and a weak positive correlation was observed between hair THg and dietary MeHg (r = 0.170).

**Conclusions:**

This study highlights the accurate estimation of MeHg intake in children using a duplicate method. Japanese children consume fish, the MeHg intakes exceeded the reference dose and/or provisional tolerable weekly intake in several children. Further discussion based on epidemiological data is required.

**Supplementary information:**

The online version contains supplementary material available at https://doi.org/10.1265/ehpm.24-00048.

## Background

Methylmercury (MeHg) is recognized to be toxic central nervous system and exhibits heightened sensitivity to the developing fetal brain in humans [1–3]. Therefore, the fetus is considered to be the highest-risk group. Advisories have been issued worldwide to women who are pregnant or may become pregnant [4]. On the other hand, although infants and children are a high-risk group considering the sensitivity of their brain development, after birth, infants and children are no longer classified into a high-risk group in Japan [4]. This is because the mercury (Hg) found in breast milk is mostly inorganic Hg [5], and MeHg is excreted by children as efficiently as in adults, and its effect on the brain is thought to be similar to that in adults [6].

In Japan, the Ministry of Health, Labour and Welfare (MHLW) recommends using lean fish from 9 months of age to avoid iron deficiency [7]. Lean fish that are eaten frequently in Japan include tuna, bonito, yellowtailm, horse mackerel, and sardines. Among them, large fish, such as tuna, are often used in baby foods and toddler meals because which can be easily deboned and safely fed to infants and children; however, MeHg tends to accumulate in higher concentrations in large fish. Infants and children are highly susceptible to MeHg because of their low body weight and high food consumption per kilogram of body weight, which can result in exposure levels of contaminants that may exceed those in adults. However, the amount of MeHg ingested by infants and children in Japan remains unclear.

There has been limited study on the degree to which infants and children consume MeHg through baby foods and toddler meals as a contributing factor to the confusion. Thus, this study aimed to determine MeHg exposure from dairy intake in baby foods and toddler meals and its correlation with total Hg (THg) levels in child hair. Methods for quantifying infants and children’s THg and/or MeHg intake include estimation using a parental food frequency questionnaire [8], and measuring THg and/or MeHg concentrations in school lunches [9] or commercial baby foods [10–13]. One of the accurate ways to determine the intake of contaminants, such as Hg, is a duplicate diet method [14]. Only studies that used the 24-hour duplicate diet method to measure Hg intake were identified in China [15] and Germany [16]. However, it should be noted that these studies only measured THg levels. Ma et al. reported that the diet primary contributes to Hg exposure among adult Japanese women, accounting for 99.7% of the total [17]. It has been reported that rice consumption is the main source of MeHg exposure in areas near mercury mines in China [18, 19], but according to data from the Pharmaceutical Affairs and Food Sanitation Council held by the MHLW, 84.2% of Japanese Hg intake comes from fish and shellfish [20]. In other words, fish is the primary source of MeHg in Japan. Thus, it is essential to determine the MeHg intake in infants or children who consume fish as baby foods or toddler meals. To this end, we employed a 24-hour duplicate diet method to collect baby foods or toddler meals samples over 3 days to measure the MeHg levels in baby foods or toddler meals and evaluate their intake. On the first day of food collection, hair samples were taken and THg levels were measured to examine their relationship with the Hg concentration in the diet.

## Methods

### Study design and participants

Dietary samples were collected from infants and children between 0–5 years of age residing in the Pacific side of the Tohoku region of Japan from Oct 2020 to June 2022 using the 24-hour duplicate diet method. Participants were recruited through various methods including flyers, newspaper advertisements, and posters placed in local government offices and hospitals. Infants and children with serious illnesses or food allergies, those on special diets owing to preterm birth or low birth weight, singleton and those who were vegetarians were excluded. Mothers whose native language was not Japanese were excluded.

**Fig. 1:**
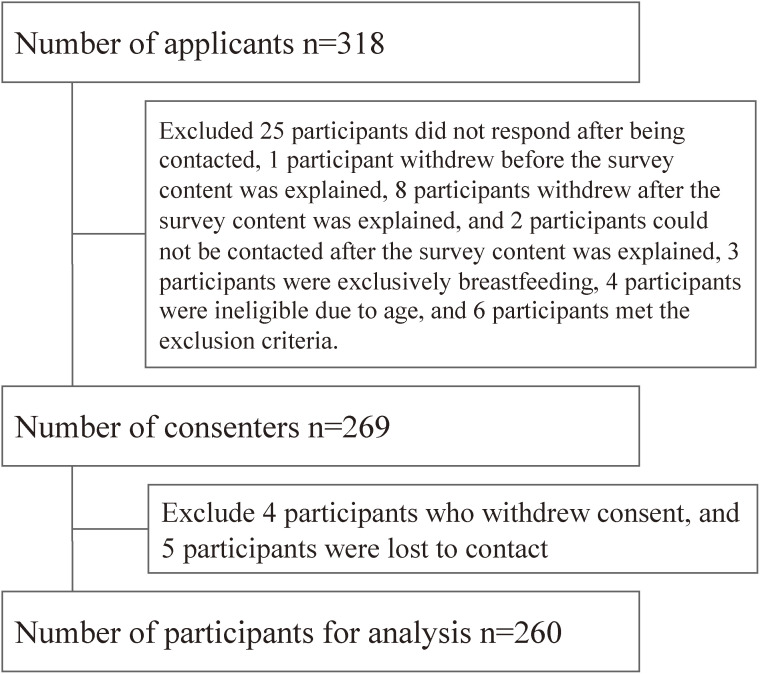
Flowchart of the study participants. In this study, participants were asked to collect three days’ worth of meals within one month from the date of consent. During that time, 16 children were confirmed to have changed their dietary stages. Because THg was measured at each dietary stage, the number of participants was 260, but the number of food samples was 276.

The MHLW has established guidelines for the diet of infants, which are divided into four age stages. The guidelines provide the appropriate nutritional content and quantify each stage, including baby food stages one (5–6 months), two (7–8 months), three (9–11 months), and four (12–17 months) [21]. Although there is no clear classification of stages for toddler meals, in many instances, 18–35 months are recognized as early-stage (toddler meal stage one), 36–47 months as middle stage (toddler meal stage two), and 48–71 months as late stage (toddler meal stage three). Diets were gathered across eight stages, including infants (0–4 months) who exclusively consumed formula milk. Thirty participants were recruited to cooperate in each dietary stage. A total of 318 participants applied, 269 participants gave informed consent, and 260 participants provided food and hair samples. The flow chart is shown in Fig. 1. The characteristics of participants in this study are shown in Table 1. Within one month of the initial sample provision, 16 infants and children changed their dietary stages. The study divided 16 participants into two stages and measured their Hg intake at each dietary stage. Although the number of participants was 260, the number of dietary samples was 276.

**Table 1:** Basal characteristics of mother-children pairs in this study by dietary stage

	**Formula milk**	**Baby foods**				**Toddler meals**		
		
		**Stage 1**	**Stage 2**	**Stage 3**	**Stage 4**	**Stage 1**	**Stage 2**	**Stage 3**
	**0–4 m**	**5–6 m**	**7–8 m**	**9–11 m**	**12–17 m**	**18–35 m**	**36–47 m**	**48–71 m**

	**Mean ± SD or number (%)**							
Number of samples	32	30	38	38	35	33	33	37
Maternal characteristics								
Maternal age	32.9 ± 4.6	33.4 ± 4.5	31.6 ± 5.5	31.8 ± 5.5	33.6 ± 4.8	33.6 ± 5.7	34.5 ± 4.1	35.3 ± 6.0
Maternal educational level (%, ≥13 years)	25 (78.1)	22 (73.3)	27 (73.0)	26 (68.4)	31 (83.8)	28 (80.0)	27 (81.8)	24 (72.7)
Annual household income (million JPY)								
≦3	0 (0.0)	4 (15.4)	6 (18.8)	3 (8.6)	2 (5.9)	1 (3.3)	2 (7.7)	4 (13.8)
3.01–5	7 (22.6)	9 (34.6)	5 (15.6)	12 (34.3)	3 (8.8)	9 (30.0)	4 (15.4)	6 (20.7)
5.01–7	14 (45.2)	9 (34.6)	15 (46.9)	16 (45.7)	15 (44.1)	15 (50.0)	12 (46.2)	14 (48.3)
7.01–10	6 (19.4)	4 (15.4)	8 (25.0)	3 (8.6)	11 (32.4)	4 (13.3)	5 (19.2)	5 (17.2)
≧10	4 (12.9)	4 (15.4)	4 (12.5)	4 (11.4)	5 (14.7)	2 (6.7)	5 (19.2)	4 (13.8)

Child characteristics								
Child age (months)	2.1 ± 1.1	5.7 ± 0.6	7.2 ± 0.8	9.8 ± 1.0	13.4 ± 2.6	25.8 ± 6.0	40.0 ± 4.0	58.3 ± 8.0
Child sex (%, boys)	18 (56.3)	10 (33.3)	16 (42.1)	22 (57.9)	18 (48.6)	14 (40.0)	18 (54.5)	16 (48.5)
Child weight (kg)	5.3 ± 0.9	7.5 ± 0.8	7.9 ± 0.8	8.6 ± 0.8	9.2 ± 1.2	12.1 ± 1.5	14.9 ± 2.0	17.6 ± 2.5
Birth order (%, ≧1)	15 (46.9)	18 (60.0)	21 (55.3)	20 (52.6)	19 (51.4)	19 (54.3)	12 (37.5)	11 (34.4)
Delivery type (%, caesarean section)	10 (31.3)	6 (20.0)	5 (13.2)	7 (18.4)	10 (27.0)	9 (25.7)	8 (24.2)	11 (33.3)
Second-hand smoke (%, yes)	10 (31.3)	11 (36.7)	11 (28.9)	13 (34.2)	6 (16.2)	14 (40.0)	9 (37.3)	9 (27.3)

Written informed consent was obtained from all participants. The study protocol was reviewed and approved by Tohoku University Graduate School of Medicine (Nos. 2020-1-205, approved on 22 June 2020 and 2021-1-108, approved on 19 May 2021) and the National Institute for Environmental Studies (NIES, IRB No. 2020-018, approved on 12 April 2021).

### Food and hair sampling and preparation

Duplicate diet studies were conducted according to the World Health Organization (WHO) Guidelines [22]. The dietary duplicate samples of children collected were all meals, including drinking water and supplements, except for prescription medications, for 3 days within a month. Duplicate portions of all consumed foods and beverages were collected in pre-cleaned polypropylene containers. Dietary samples in polypropylene containers were stored in a household refrigerator until they were sent to the laboratory, where they were stored with refrigerants. Samples were homogenized for each collection day, divided proportionally by the ratio of the sample quality weight for each day to the total wet weight for the 3 days, and mixed to obtain the required volume for analysis. The samples were lyophilized and homogenized in powder form to obtain the samples for THg and MeHg measurements.

Following the Manual for Mercury Analysis [23], samples were acid digested and analyzed by cold vapor atomic absorption spectrometry (CVAAS, Hg-201, Sanso Inc, Tokyo, Japan). For quality control, the reference materials of skimmed milk powder (ERM BD-150) purchased from the European Commission and wheat flour (SRM1567b) from the National Institute of Standards and Technology (MD, USA) were measured. The THg recovery for ERM BD-150 and SRM1567b were 88–92% (N = 9) and 96–130% (N = 9), respectively.

For the measurement of MeHg, the samples were alkaline-decomposed and extracted with dithizone toluene, and the cleaned samples were measured using a gas chromatography-electron capture detector (GC-ECD, G2700, Yanaco Analytical Systems Inc., Kyoto, Japan). For quality control, reference materials for fish protein powder (DORM-4) were purchased from the National Research Council. The MeHg recovery for DORM-4 was 98–102% (N = 3). Method detection limit (MDL) and method quantification limit (MQL) were calculated based on the standard deviation of seven replicated analyses of standard samples with low Hg levels. The MDL and MQL of THg for CVAAS were 0.03 and 0.08 ng, respectively. The MDL and MQL of MeHg for GC-ECD were 0.18 and 0.45 ng, respectively. THg and MeHg in dietary samples were analyzed by IDEA Consultants, Inc. (Tokyo, Japan). In this study, concentration below MDL were substituted by half the MDL value.

Hair samples were collected from the occipital area of each child on the first day of diet sample collection. The hair used for THg analysis was 3 cm from the root of the occiput. The concentration of THg in hair was measured using the direct thermal decomposition-gold amalgamation-CVAAS (MA-3000, Nippon Instruments Corporation, Osaka, Japan). To ensure reliability, hair reference materials NIES No.13 (National Institute for Environmental Studies, Tsukuba, Japan) and IAEA-086 (International Atomic Energy Agency, Vienna, Austria) were also analyzed using the CVAAS for THg. The mean (RSD) of hair analysis for NIES No.13 and IAEA-086 were 4.43 ng/mg (1.8%) and 0.576 ng/mg (2.3%), which were within the certified values (N = 30, inter-day).

### Basic characteristics

Information regarding maternal age, level of maternal education, annual income, second-hand smoke (yes or no), child sex (sex assigned at birth), birth order, weight, and delivery type was collected using a questionnaire. In relation to second-hand smoke, if a family member who lives with the child in question smokes, answers were given from three options: a) Completely separated smoking, b) Partially separate smoking, and c) No separate smoking. If the answer was a), it was rated as “no,” and if the answer was or b) or c), it was rated as “yes.” Mothers were asked about their children’s consumption of fish on a five-point scale. scale: ‘never,’ ‘less than once a month,’ ‘more than once a month,’ ‘at least once a week,’ and ‘more than once a day.’ The mothers maintained a food diary that recorded the ingredients used in their meals and information on any commercial products consumed, including the manufacturer and ingredients.

## Results

Table 2 shows the concentrations of THg in the dietary samples, and the amount of the intake (g). A considerable proportion of diet samples, particularly those from formula milk and baby food stages one and two, showed THg levels ≤MDL and ≤MQL. The median THg concentration in the diet was 0.34 (95% confidence interval (CI), 0.02–3.53) ng/g (wet). The estimated daily intake of THg was 20.3 (0.72–232.5) ng/kgbw/day. Dietary intake and child weight increased with age; however, THg intake is higher during baby food stages three and four.

**Table 2:** THg in the diet samples

	**n**	**MDL**		**MQL**		**THg** **(ng/g-wet)**			**Amount** **(g)**			**THg intake** **(ng/kgbw/day)**		
				
		**n**	**%**	**n**	**%**	**Median**	**5–95%tile**		**Median**	**5–95%tile**		**Median**	**5–95%tile**	
Formula milk														
	32	20	62.5	10	31.3	0.020	0.015	0.099	495.1	134.7	905.8	2.4	0.5	6.9
Baby foods														
Stage 1	30	4	13.3	12	40.0	0.085	0.017	0.974	371.4	24.7	980.6	4.2	0.5	14.0
Stage 2	38	5	13.2	10	26.3	0.150	0.020	2.905	474.9	46.0	1032.5	7.0	0.6	103.3
Stage 3	38	0	0.0	0	0.0	0.445	0.181	5.500	656.9	209.6	1030.6	25.0	8.5	492.8
Stage 4	35	0	0.0	3	8.6	0.500	0.134	3.620	772.9	257.7	1236.3	41.0	7.9	329.6
Toddler meals														
Stage 1	33	0	0.0	1	3.0	0.510	0.191	3.630	847.7	410.9	1242.7	32.4	11.0	259.9
Stage 2	33	0	0.0	1	3.0	0.490	0.204	4.260	941.6	508.6	1277.6	36.0	9.5	252.9
Stage 3	37	0	0.0	2	5.4	0.450	0.192	1.900	1073.7	642.4	1666.3	25.2	8.4	125.6

MDL, method detection limit; MQL, method quantification limit; THg, total mercury

Concentration below MDL were substituted by half the MDL value.

MeHg was not detected in the diet when the dietary THg concentration was <1 ng/g (Table 3). Since the MDL of MeHg is 0.2 ng/g, even assuming that MeHg in the diet is 50% of THg, it makes sense that MeHg would not be detected if THg is <1 ng/g. There were 63 samples (22.8%) with dietary THg concentrations of 1 ng/g or higher. In these samples, MeHg analysis revealed a median value of 1.70 (0.87–6.21) ng/g, with MeHg accounting for 90.0% of THg (Table 3). MeHg concentrations were higher in baby food stages one, two, and three. After multiplying the MeHg concentration by the intake amount and dividing it by the child weight, it was found that MeHg intake was high in baby food stages three and four and gradually decreased after that. The MeHg intake was estimated by multiplying the THg concentration in all samples by 90% (MeHg as a percentage of THg). The median estimated MeHg intake for all participants was 18.3 (0.65–209.2) ng/kgbw/day. Table 4 shows the estimated MeHg intake for each dietary stage. MeHg intake was highest in baby food stages three and four, with a subsequent downward trend.

**Table 3:** MeHg in the diet samples with dietary THg concentrations ≥1 ng/g (n = 63)

	**≥1 ng/g**		**MeHg** **(ng/g-wet)**			**% (MeHg/THg)**			**MeHg intake** **(ng/kgbw/day)**		
		
	**n**	**%**	**Median**	**5–95%tile**		**Median**	**5–95%tile**		**Median**	**5–95%tile**	
Formula milk											
	0	0	—	—	—	—	—	—	—	—	—
Baby foods											
Stage 1	2	6.7	2.60	1.16	4.04	91.1	90.9	91.3	38.0	6.2	69.8
Stage 2	6	15.8	2.55	0.95	3.68	85.7	67.4	96.4	26.7	13.6	171.1
Stage 3	12	31.6	2.05	0.97	6.75	92.0	76.1	97.2	160.7	46.9	625.6
Stage 4	16	43.2	1.55	0.96	3.63	88.7	73.1	98.0	163.2	64.8	346.6
Toddler meals											
Stage 1	11	31.4	1.40	0.92	6.25	91.7	80.0	96.8	98.4	35.8	420.7
Stage 2	10	30.3	1.65	0.94	6.52	87.9	77.5	94.9	119.3	30.4	367.6
Stage 3	6	18.2	1.60	0.84	2.50	91.6	69.5	99.1	107.3	38.7	192.5

MeHg, methylmercury; THg, total mercury

**Table 4:** Estimated dietary intake of MeHg (n = 276)

	**THg (ng/g-wet) × 90%**			**Estimated MeHg intake (ng/kgbw/day)**		
	
	**Median**	**5–95%tile**		**Median**	**5–95%tile**	
Formula milk						
	0.02	0.01	0.09	2.2	0.5	6.2
Baby foods						
Stage 1	0.08	0.02	0.88	3.8	0.4	12.6
Stage 2	0.14	0.02	2.61	6.3	0.5	93.0
Stage 3	0.40	0.16	4.95	22.5	7.6	443.5
Stage 4	0.45	0.12	3.26	36.9	7.1	296.6
Toddler meals						
Stage 1	0.46	0.17	3.27	29.2	9.9	233.9
Stage 2	0.44	0.18	3.83	32.4	8.5	227.6
Stage 3	0.41	0.17	1.71	22.7	7.5	113.0

MeHg, methylmercury; THg, total mercury

**Fig. 2:**
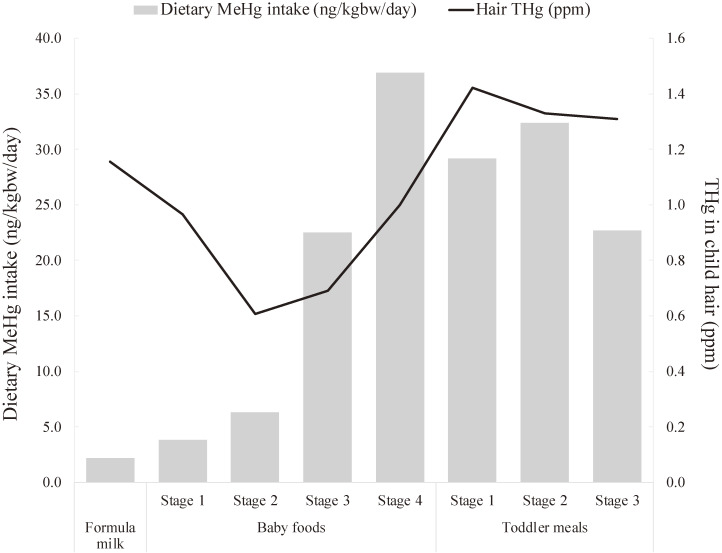
Dietary MeHg intake and hair THg concentration of children THg, total mercury; MeHg methylmercury

The median THg value in infants and children’s hair was 1.1 (0.3–4.0) ppm. The median hair THg level was >1 ppm in stage one formula milk and baby food stage one, followed by a decrease in THg concentration from stages two to three and an increase from stage three baby food to stage one toddler meal (Table 5, Fig. 2). Table 6 shows the correlation coefficients between dietary MeHg and hair THg. A negative association was observed between hair THg and dietary MeHg because the dietary MeHg concentration was low from baby formula to baby food stage two. The correlation coefficients between MeHg intake in the diet and THg levels in hair for baby food stage four and toddler meal stages one and two were moderately correlated, with values ranging from 0.3 to 0.4.

**Table 5:** Hair THg results by dietary stage (ppm)

	**Median**	**5–95%tile**		**over 1.0 ppm** **(over RfD)**		**over 2.2 ppm** **(over PTWI)**	
	
				**n**	**%**	**n**	**%**
Formula milk	1.2	0.4	3.9	19	59.4	5	15.6
Baby foods							
Stage 1	1.0	0.3	2.5	14	46.7	2	6.7
Stage 2	0.6	0.2	1.4	9	23.7	1	2.6
Stage 3	0.7	0.3	1.9	13	34.2	0	0.0
Stage 4	1.0	0.3	4.4	18	51.4	7	20.0
Toddler meals							
Stage 1	1.4	0.6	2.8	26	78.8	7	21.2
Stage 2	1.3	0.3	6.0	21	63.6	10	30.3
Stage 3	1.3	0.4	4.5	23	62.2	11	29.7

Total	1.1	0.3	4.0	143	51.8	43	15.6

THg, total mercury; RfD, reference dose; PTWI, provisional tolerable weekly intake

RfD and PTWI were converted to hair THg

**Table 6:** Correlation coefficient between dietary MeHg intake and hair THg

	**r value**	**p value**
Formula milk	−0.403	0.030
Baby foods		
Stage 1	−0.218	0.246
Stage 2	−0.083	0.622
Stage 3	0.184	0.270
Stage 4	0.493	0.002
Toddler meals		
Stage 1	0.343	0.044
Stage 2	0.401	0.021
Stage 3	0.162	0.369

THg, total mercury; MeHg, methylmercury

Supplementary Table 1 shows the relationship between hair THg and estimated MeHg intake with basal characteristics. There were no statistically significant differences observed in hair THg levels or estimated MeHg intake based on annual income, child sex, or region of residence. Although there were no statistically significant differences, children residing in coastal areas had higher hair THg and estimated MeHg intakes compared to children residing in urban or rural areas. Supplementary Table 2 shows the difference in hair THg and estimated MeHg intake according to the frequency of fish intake. The relationship between frequency of fish intake and region of residence was also shown. No differences were observed between the frequency of fish intake and hair THg. However, as the frequency of fish intake increased, the estimated intake of MeHg also increased (p < 0.001). Additionally, fish intake was more frequent in the coastal area.

## Discussion

This is the first study to measure MeHg levels in 3-day collections of diets consumed by infants and children aged 0–5 years, using the duplicate diet method to establish postnatal MeHg intake. Many specimens from formula milk and baby food at stages one and two were found to have undetectable THg levels. MeHg concentrations were higher in baby food stages one to three than in subsequent food stages. However, when converted by infants and children’s body weight, MeHg intake was higher in baby food stages three to four and then decreased. The percentage of MeHg in the dietary THg of Japanese infants or children was 90%. The high proportion of MeHg in the diet may have been due to the fact that Japanese people, even infants or children, consume a lot of fish.

To the best of our knowledge, no studies have examined MeHg intake in infants and children using the 24-hour duplicate diet method. Therefore, we compared our THg results to those of previous studies. The THg median weekly intake in this study, which was 0.14 µg/kgbw/week in our study, was almost identical to the 0.13 µg/kgbw/week found in Germany [16]. However, compared to the THg range of our study (0.01–1.63 µg/kgbw/week, n = 276), the German study had a narrower range (0.060–0.62 µg/kgbw/week, n = 14), probably due to the small sample size. In a previous Chinese study, 66 children who did not consume fish had a THg intake of 0.91 (0.35–5.46) µg/kgbw/week, while 22 children who ate fish had an intake of 1.12 (0.77–5.88) µg/kgbw/week [15]. Overall, the measured THg intake of Japanese infants and children was comparable to or slightly higher than that of German children and lower than that of Chinese children. According to data calculated using the 24-hour duplicate diet method, the average THg intake of adult women in Japan was 0.504 ± 0.98 µg/kgbw/week (n = 37) [17]. In the study conducted in China, children had a higher intake of THg than adults because of their lower body weight [15]. However, the present study revealed that infants or children had lower THg intake than adults. It is important to note that the two studies had different target populations; the Chinese study focused on children aged 2–7 years, while the present study targeted children aged 0–5 years. The variation in age range may be the reason for the difference in results.

The reference dose (RfD) of MeHg was established by the US Environmental Protection Agency in 1995 [24]. Its purpose was to prevent adverse effects in high-risk groups, especially pregnant women, and MeHg-sensitive fetuses. The established RfD is 0.1 µg/kgbw/day. In this study, 38 infants or children (13.8%) had MeHg intake that exceeded the RfD. A higher percentage of infants or children exceeded the RfD at baby food stages three and four (Supplementary Table 3). The 61st FAO/WHO Joint Expert Committee on Food Additives meeting in June 2003 revised the provisional tolerable weekly intake (PTWI) from 3.3 µg/kgbw/week established in 1972 to 1.6 µg/kgbw/week [25]. Twelve infants or children (4.3%) had MeHg intake exceeding the PTWI of 1.6 µg/kgbw/week. Further studies are warranted to clarify the impact of MeHg exposure from baby foods and toddler meals on infants or children who exceed the PTWI [25].

Our findings indicate that MeHg intake increases during baby food stages three to four and then declines during the toddler meal stages (Fig. 2). The proportion of infants or children exceeding the PTWI as well as the RfD was higher during baby food stages three to four (Supplementary Table 3). In Japan, the MHLW has published a guide for baby foods, in which it is recommended to use red meat fish in the diet from the age of 9 months onwards, because iron deficiency is easily observed [7]. Iron deficiency is a serious problem after 6 months of age so there was an opinion that it would be better to start consuming red meat fish at 6 months [26], but the current guidelines issued by the MHLW have not changed from 9 months [7]. This could explain the increase in MeHg intake during baby food stages three to four. The common types of red meat fish consumed in Japan include tuna, swordfish, bonito, and canned tuna. Red meat fish with high MeHg content, such as tuna, are often preferred because they are boneless, easy to cook, and safe for infants and children to eat without worrying about fish bones. Thus, it is likely that the MeHg intake increased during this period. From a nutritional perspective, it is being recommended to start consuming red meat fish earlier, but from the perspective of protecting children, we would like them to consider the issue of exposure to chemicals, such as MeHg. The MeHg intake decreased slightly when transitioning to toddler meal. The transition from baby foods to toddler meal allows infants and children to consume a wider range of foods, possibly leading to decreased MeHg intake because these foods contain lower levels of MeHg. Consuming fish provides nutritional benefits, as it enables the intake of iron, high-quality protein, abundant vitamins, and polyunsaturated fatty acids [27, 28]. It is crucial to avoid overeating certain types of fish and to strive for a balanced diet.

Hair samples were collected from children aged 0–5 years and analyzed for THg levels. The median THg concentration in the hair of formula milk stage children (0–4 months) was 1.2 ppm (0.4–3.9), followed by a gradual decrease, and an increase around the baby food stage four (12–17 months). As previously mentioned, the RfD for MeHg is 0.1 µg/kgbw/day, and PTWI is 1.6 µg/kgbw/week. Converting this intake to hair THg values would equate to 1.0 µg/g for RfD and 2.2 µg/g for PTWI [29]. This study observed that 143 infants or children (51.8%) had hair THg levels above 1.0 µg/g, with 43 infants or children (15.6%) exceeding 2.2 µg/g (Table 5). When examining the percentage of infants or children exceeding the RfD and PTWI for each diet stage, a pattern emerged with high initial levels, followed by a decline, a subsequent increase, and a final decrease. Previous studies have indicated a positive correlation between hair THg levels and fish consumption [30, 31]. A study targeting children aged 5–17 years reported a correlation of r = 0.291 between fish intake and hair THg level (n = 94) [32]. Thus, we hypothesized that MeHg intake would correlate with hair THg levels. However, significant positive correlations between hair THg and MeHg intakes were observed only at certain dietary stages (Fig. 2). Among the eight dietary stages, formula milk and baby food stages one and two negatively correlated with hair THg levels. Hair THg is thought to increase immediately after delivery due to the transfer of MeHg from the placenta to the fetus during the fetal period. In contrast, formula milk and baby food stages one and two contained little MeHg (Table 2), which may have decreased due to the low MeHg intake from formula milk and baby food stages one and two. As MeHg intake increased in baby food stages three and four, hair THg level increased. MeHg intake decreased slightly in the toddler meal stage, and hair THg levels tended to decrease. This indicates that MeHg intake and hair THg levels are not necessarily correlated in infants and children. Miklavčić et al. indicated that hair THg levels serve a valuable purpose as a biomarker for MeHg exposure from consuming fish [33], but it is important to use discretion when interpreting data from infants and children due to MeHg placental transfer during the fetal period.

The strength of our study is the use of a 24-hour duplicate method to collect three days’ worth of meals, the measurement of not only THg but also MeHg in children’s meals, and the estimation of intake for each dietary stage. However, this study has several limitations. First, this study was limited to children residing in the Tohoku region of Japan due to movement restrictions in place to prevent the spread of COVID-19 during the survey period. To ensure a highly representative group, recruitment activities were conducted through newspaper advertisements and a wide range of local governments and hospitals. Although the survey was limited to the Tohoku region, we were able to collect data from coastal, rural, and urban areas. Since the purpose of this study is to estimate MeHg intake in children, sample size extrapolation cannot be predicted. Although this study had the largest number of participants among the studies that estimated MeHg intake using the duplicate method, it was not possible to demonstrate whether the sample size was statistically sufficient. Second, studies on Japanese adults have indicated that hair grows at approximately 1.3 cm/month [34], but the growth rate of hair in infants and children is unknown. Thus, interpretating the correlation between dietary MeHg and hair THg was requires careful consideration. Furthermore, the WHO recommends a Hg hair-to-blood ratio of 250 for converting THg hair levels to those in whole blood [35]. However, the infant-child ratio remains unknown. Further studies analyzing the associations between MeHg intake and exposure biomarkers in hair and blood samples will facilitates a comprehensive understanding of the MeHg exposure status in infants or children.

## Conclusions

Over the course of this study, a 24-hour duplicate diet method was used to collect 3 days of diets to measure MeHg intake among Japanese infants aged 0–5 years. MeHg intake increased between approximately 9 months and 3 years of age, after which it decreased slightly. During this period, it was confirmed that the infants exceeded the RfD and PTWI. Although considerable research has been conducted on the health impacts of prenatal MeHg exposure, the effects of postnatal MeHg exposure in infants remain unclear. Further studies are required to assess the health effects of MeHg intake in infants.

## Abbreviations

MeHg: methylmercury
Hg: mercury
THg: total mercury
CVAAS: cold vapor atomic absorption spectrometry
MDL: method detection limit
MQL: method quantification limit
GC-ECD: gas chromatography-electron capture detector
CI: confidence interval
RfD: reference dose
PTWI: provisional tolerable weekly intake
MHLW: Ministry of Health, Labour and Welfare

## Supplementary information

**Additional file 1:** **Supplementary Table 1** Basal characteristics and hair THg/estimated MeHg intake. **Supplementary Table 2** Residence area and hair THg/estimated MeHg intake due to differences in fish intake. **Supplementary Table 3** Number of children who exceeded RfD/PTWI.
