# Supplementary material for: Dietary intake of methylmercury by 0–5 years children using the duplicate diet method in Japan
Source: Environ Health Prev Med. 2024 May 10;29:27. doi: 10.1265/ehpm.24-00048 (PMC11111292; doi:10.1265/ehpm.24-00048)
Supplement: Supplementary file 1 — Additional file 1: Supplementary Table 1 Basal characteristics and hair THg/estimated MeHg intake. Supplementary Table 2 Residence area and hair THg/estimated MeHg intake due to differences in fish intake. Supplementary Table 3 Number of children who exceeded RfD/PTWI. [file ehpm-29-027-s001.docx]

| Supplementary Table 1 Basal characteristics and hair THg/estimated MeHg intake | | | | | |
| --- | --- | --- | --- | --- | --- |
|  |  |  | Hair THg  (ppm) |  | Estimated MeHg intake (ng/kgbw/day) |
|  |  |  | Mean±SD | | |
| Annual income | | | |  |  |
|  | ≦ 3 | n=22 | 1.2±0.8 |  | 15.3±13.3 |
|  | 3.01-5 | n=55 | 1.2±1.0 |  | 52.3±116.1 |
|  | 5.01-7 | n=110 | 1.4±1.3 |  | 57.2±101.3 |
|  | 7.1-10 | n=46 | 1.4±1.2 |  | 31.9±37.1 |
|  | ≧10 | n=32 | 1.4±1.2 |  | 53.7±64.5 |
|  | F value | | 0.265 |  | 0.696 |
|  | p value | | 0.901 |  | 0.595 |
| Child sex | |  |  |  |  |
|  | Male | n=144 | 1.4±1.3 |  | 57.9±105.5 |
|  | Female | n=132 | 1.3±1.1 |  | 40.6±67.6 |
|  | F value | | 0.668 |  | 0.635 |
|  | p value | | 0.414 |  | 0.426 |
| Residence area | | | |  |  |
|  | urban area | n=103 | 1.2±1.1 |  | 38.7±51.1 |
|  | rural area | n=50 | 1.2±0.8 |  | 47.6±76.0 |
|  | coastal area | n=123 | 1.6±1.5 |  | 59.7±115.8 |
|  | F value | | 2.556 |  | 0.645 |
|  | p value | | 0.079 |  | 0.525 |
| THg, total mercury; MeHg, methylmercury | | | | |  |

| Supplementary Table 2 Residence area and hair THg/estimated MeHg intake due to differences in fish intake | | | | | | | | | | | | | | | | | | | |  |  |
| --- | --- | --- | --- | --- | --- | --- | --- | --- | --- | --- | --- | --- | --- | --- | --- | --- | --- | --- | --- | --- | --- |
|  |  | Never |  | Less than once a month |  | | More than once a month | |  | | At least once a week | |  | | More than once a day | |  | | p value* | |  |
|  |  | n=53 |  | n=10 |  | | n=30 | |  | | n=159 | |  | | n=24 | |  | |  |  |  |
|  |  | Mean±SD or number (%) | | | | | | | | | | | | | | | |  | | | |
| Hair THg | | 1.2±0.9 |  | 1.1±0.9 |  | | 1.0±0.7 | |  | | 1.5±1.3 | |  | | 1.8±1.5 | |  | | 0.205 | |  |
| Estimated MeHg intake | | 4.4±10.1 |  | 22.8±31.1 |  | | 22.7±38.5 | |  | | 57.4±78.6 | |  | | 142.0±189.2 | |  | | <0.001 | |  |
| Residence area | |  |  |  |  | |  | |  | |  | |  | |  | |  | | 0.019 | |  |
|  | urban | 23 (8.3) |  | 6 (2.2) |  | | 15 (5.4) | |  | | 51 (18.5) | |  | | 8 (2.9) | |  | |  | |  |
|  | rural | 13 (4.7) |  | 0 (0.0) |  | | 8 (2.9) | |  | | 23 (8.3) | |  | | 6 (2.2) | |  | |  | |  |
|  | coastal | 17 (6.2) |  | 4 (1.4) |  | | 7 (2.5) | |  | | 85 (30.8) | |  | | 10 (3.6) | |  | |  | |  |
| SD, standard deviation; THg, total mercury; MeHg, methylmercury | | | | | |  | |  | |  | |  | |  | |  | |  | |  |  |

| Supplementary Table 3 Number of children who exceeded RfD/PTWI | | | | | | |
| --- | --- | --- | --- | --- | --- | --- |
|  |  |  |  |  |  |  |
|  |  | ≥ RfD | |  | ≥ PTWI | |
|  |  | n | % |  | n | % |
| Formula milk | | 0 | 0.0 |  | 0 | 0.0 |
| Baby foods | |  |  |  |  |  |
|  | Stage 1 | 0 | 0.0 |  | 0 | 0.0 |
|  | Stage 2 | 1 | 0.4 |  | 0 | 0.0 |
|  | Stage 3 | 10 | 3.6 |  | 4 | 1.4 |
|  | Stage 4 | 11 | 4.0 |  | 4 | 1.4 |
| Toddler meals | |  |  |  |  |  |
|  | Stage 1 | 6 | 2.2 |  | 2 | 0.7 |
|  | Stage 2 | 7 | 2.5 |  | 2 | 0.7 |
|  | Stage 3 | 3 | 1.1 |  | 0 | 0.0 |
| Total | | 38 | 13.8 |  | 12 | 4.3 |
| RfD, reference dose (0.1µg/kgbw/day); PTWI, provisional tolerable weekly intake (1.6µg/kgbw/week) | | | | | | |
|  |  |  |  |  |  |  |
